# Green Turtle Conservation in the Genomic Era—Monitoring an Endangered Mediterranean Population and Its Breeding Habits

**DOI:** 10.1002/ece3.71124

**Published:** 2025-04-24

**Authors:** Talya Ohana, Larissa S. Arantes, Gili Tikochinski, Reut Cohen, Olga Rybak, Adi Gaspar, Sarah Sparmann, Susan Mbedi, Yaniv Levy, Camila J. Mazzoni, Yaron Tikochinski

**Affiliations:** ^1^ Ruppin Academic Center Faculty of Marine Sciences Emek Hefer Israel; ^2^ Leibniz Institute for Zoo and Wildlife Research (IZW) in the Forschungsverbund Berlin eV, Genetics, Ecology and Evolution Berlin Berlin Germany; ^3^ Israeli Sea Turtle Rescue Center, Israel Nature and Parks Authority Michmoret Israel; ^4^ Berlin Center for Genomics in Biodiversity Research Berlin Germany; ^5^ Museum für Naturkunde – Leibniz‐Institut für Evolutions‐ und Biodiversitätsforschung Berlin Berlin Israel

## Abstract

The Mediterranean Sea green turtle population, confined to the eastern basin, is classified as endangered by the IUCN. The small Israeli nesting population significantly contributes to the genetic variability of the Mediterranean population, underscoring the importance of Israeli shores as a key migration route. The Israeli rescue center has established the only active breeding stock in the Mediterranean, with considerable time and effort invested in its inception and operation. Here, we present a new genomic approach aimed at maximizing this population's genetic contribution to the Mediterranean. We studied 269 green turtles from both the breeding stock and the natural Mediterranean population using mitochondrial DNA short tandem repeat (STR) haplotyping and genomic double‐digest RAD sequencing. Our analysis identified multiple degrees of kinship among individuals and aided in detecting female breeding habits. We analyzed 84 nests laid along the Israeli shores over the past 20 years and identified 60 mating events involving 35 nesting females. By incorporating females identified in previous studies, we estimate that at least 51 females have nested along the Israeli coastline during this period—a number significantly higher than earlier estimates. Furthermore, at least 59 males participated in these 60 mating events, highlighting a greater‐than‐expected level of genetic diversity within this population. We confirmed documented reproductive behaviors such as sperm conservation and multiple paternity. Additionally, we determined nesting intervals both within and between years. While our results supported the well‐documented philopatric characteristics of green turtle females, they also revealed that some females laid eggs up to 90 km apart. This genomic‐assisted evaluation offers crucial insights into the genetic variability and breeding habits of the Israeli green turtle population, providing a valuable model for conservation efforts across the Mediterranean region.

## 1 Introduction

Sea turtles have been navigating Earth's oceans for more than 100 million years (Bowen and Karl 2007). The Mediterranean Sea hosts critical nesting habitats for two of the seven sea turtle species, namely the loggerhead turtle ( *Caretta caretta* ) and the green turtle ( *Chelonia mydas* ). While the loggerhead population has shown signs of recovery over the past decade (Casale 2010), the status of the Mediterranean green turtle remains precarious, with the species classified as endangered by the International Union for Conservation of Nature (IUCN 2024).

Green turtles have intricate breeding habits that are essential for the propagation of the species. These turtles are well known for their extensive migratory patterns, often traveling thousands of kilometers from feeding grounds to nesting sites (Lohmann and Lohmann 2019; Godley et al. 2002; Lutz et al. 2003). Green turtle females exhibit strong philopatry, consistently returning to the same nesting beaches where they were born to mate and lay eggs. This behavior is believed to enhance reproductive success by ensuring that they nest in environments that support their own survival as hatchlings (Bowen and Karl 2007).

Green sea turtles typically reach sexual maturity between 20 and 50 years of age. During the breeding season, which usually coincides with warmer months, males and females gather near nesting beaches and mating occurs in the water, normally close to shore. Female green turtles generally nest every 2–4 years. During a nesting season, a single female may mate with several males, store sperm, and lay multiple clutches of eggs, with intervals of about 2 weeks between each nesting event. On average, a female lays between three and five clutches per season, though some may lay up to seven (Hirth 1980; Lutz et al. 2002; Spotila 2004).

Green turtle nesting in the Mediterranean is confined to the eastern basin, from eastern Turkey through Cyprus, which hosts over 90% of the nests, to eastern North Africa (Egypt) (Casale 2010; Casale et al. 2018; Karaman et al. 2022; Türkozan et al. 2013). Although the number of nesting females was once estimated to be 1350 across the region (Stokes et al. 2015), the actual number of females and males remains unknown.

The Israeli shores host a very small fraction of the Mediterranean nesting population, with about 20–30 nests laid each year (Levy and Rybak 2022). Based on a long‐term conservation management program of nest surveys along the coastline, the Israeli Nature and Parks Authority (INPA) estimated that only 10–12 females nest along the Israeli shores (Kuller 1999). In 2002, the INPA rescue center launched a green turtle breeding program to recuperate the highly threatened population nesting along the Israeli coastline. Hatchlings from two different nests, one in the south of Israel (Ashkelon Nature Park) and one in the north (Gdor Nature Reserve), were brought to the rescue center where most of them lived until 2019. In 2019, they were transferred to a new rescue center facility, where they now reside in two separate ponds with artificial nesting grounds. Females from each of the two families inhabit the two different ponds, along with males from the opposite family.

Over the past three decades, genetic markers like Single Nucleotide Polymorphisms (SNPs) in the mitochondrial DNA control region (D‐loop) have been used to study the green turtle population globally. However, this approach was less effective for the Mediterranean population due to the predominance of the CM‐A13 mtDNA D‐loop haplotype (Bowen et al. 1992; Kaska 2000; Bagda et al. 2012). To complement these limitations, microsatellite markers—or nucleotide short tandem repeats—have been widely employed to investigate sea turtle population structure. These markers have furthered our understanding of critical behaviors such as natal homing and reproductive strategies, offering insights into gene flow, genetic diversity, and connectivity among populations (Wright, Fuller, et al. 2012; Wright, Stokes, et al. 2012; Barbanti et al. 2022; Dolfo et al. 2023).

A novel mtDNA haplotyping method, using sequences from the mtDNA D‐loop 3′ region, which includes AT tandem repeats, has advanced the study of Mediterranean green turtles (Tikochinski et al. 2012). This method revealed 10 haplotypes among Israeli nesting females and 33 haplotypes when including stranded turtles. The study also found different haplotypes in the two breeding stock families, suggesting unrelated mothers.

A broader follow‐up study analyzing 517 samples from nine Mediterranean sites identified 37 mtDNA haplotypes. Sixteen of these haplotypes were shared by Israeli nesting females, and 17 of 21 “non‐Israeli” haplotypes were found in stranded turtles, underscoring Israeli shores as a key migration route (Tikochinski et al. 2018). This study identified four distinct management units, including the unique Israeli shore population. Recently, 11 additional mtDNA haplotypes were discovered in the northern Mediterranean population (Karaman et al. 2022). Expanding the survey of the Israeli population revealed that approximately 74% of all identified haplotypes are present along the Israeli shores, as reported in the present study.

Since these studies focused on maternally inherited mtDNA, they only reveal females' contributions to genetic variability. Furthermore, the high abundance of certain mtDNA haplotypes prompted the identification of reliable nuclear markers to differentiate between females with the same haplotype.

Advancements in high‐throughput sequencing have enabled cost‐effective genome‐wide assessments. New methods allow the survey of genetic markers across many individuals (Harrisson et al. 2014), providing insights into species' life histories and population dynamics (Davey et al. 2011). An important innovation in NGS is restriction site‐associated DNA sequencing (RADseq), which isolates thousands of nuclear markers for population genomic analyses (Catchen et al. 2013). RADseq's versatility has expanded with double‐digest RADseq (ddRADseq), enhancing marker density and reproducibility (Arantes et al. 2023; Peterson et al. 2012). When a reference genome is available, RADseq can map reads directly to the reference, improving SNP detection accuracy and reducing biased analysis due to linkage disequilibrium.

Over a decade ago, Wang et al. (2013) assembled the first draft of the green turtle genome, marking a significant milestone in understanding turtle evolution. Recently, the genomic sequencing of a male from the Israeli breeding stock led to the publication of an improved version of this genome, which has been instrumental in exploring evolutionary aspects of sensory and immune genes in sea turtles (Bentley et al. 2023).

In this study, we analyzed 269 samples of the Israeli green turtle population, employing both the conventional mitochondrial DNA short tandem repeat (STR) haplotyping and the innovative ddRADseq approach to showcase how advancements in genomic research can enhance the assessment of genetic variability and breeding habits in green turtles. We aimed to illustrate the determination of kinship and relatedness by utilizing thousands of SNPs. This extensive array of markers provides an understanding of the actual contribution of different nesting females to the local population. It also allows for tracking the breeding habits of green turtle females, such as natal homing, polyandry, and nesting intervals. Additionally, understanding the Mediterranean male contribution to the Israeli green turtle gene pool becomes feasible. Given that a breeding stock program has already commenced, we aimed to establish the genomic foundation necessary to support its goal of not only increasing the population size but also maintaining and enhancing its genetic variability.

## 2 Materials and Methods

### 2.1 Sample Collection and DNA Extraction

269 green turtle ( *C. mydas* ) samples were obtained from the Israeli captive breeding stock and natural populations. Table S1 lists all adult and hatchling samples participating in this study, and Figure S1 is a map of their locations. DNA from the 22 breeding stock members and 10 other adults was obtained from blood samples between 2002 and 2022. DNA was also obtained from 237 dead hatchlings sampled in 84 different nests along the shores of Israel during that period, representing about 5% of the nests laid during the study period. For 18 of these nests, we obtained five samples or more to assess multiple paternity. Blood sampling was permitted by the Israeli Nature and Park Authority (NPA) and was taken as part of routine health checks. According to the manufacturer's protocol, DNA was extracted from blood or tissue samples by the DNeasy Blood & Tissue Kits (Qiagen). Accurate concentration measurements of DNA samples were made using the Quant‐iT PicoGreen dsDNA Assay. All samples were normalized to a concentration of 20 ηg/μL.

### 2.2 mtDNA Haplotyping

An approximately 250 bp fragment of the AT‐rich 3′ end of the mtDNA control region was amplified using the primer pair CM‐D‐1F (5′‐AGCCCATTT ACTTCT CGCCAAACCCC‐3′) and CM‐D‐5R (5′‐GCTCCTTTTATCTGATGGG ACTGTT‐3′) (Tikochinski et al. 2012). Polymerase chain reactions (PCRs) were carried out in 20 μL and contained 1X DreamTaq PCR Master Mix (Thermo Scientific), 0.5 μΜ of each primer, and about 40 ηg template DNA. PCR products were visualized by electrophoresis to ensure successful amplification. The mtDNA amplicons were sequenced in forward and reverse directions in an ABI 3730 DNA Analyzer (Applied Biosystems). Sequences were edited and aligned using BIOEDIT 7.2.5 (Hall 1999). Short tandem repeats (STRs) were scored by counting the number of “AT” repeats in each of the four tandem repeat loci of the sequence, and haplotypes were defined by combining the four STRs and named using the four‐number barcoding system described in the literature (Tikochinski et al. 2012). In cases of mtSTRs heteroplasmy, the major haplotype was taken, based on the relative peak heights, as previously described (Tikochinski et al. 2018).

### 2.3 ddRAD Laboratory

Double‐digest RADseq (ddRAD) libraries were produced using the 3RAD protocol of Hoffberg et al. (2016). Briefly, 10 μL of genomic DNA (200 ng) were digested with three 6‐cutter enzymes: EcoRI‐HF, XbaI, and a third adapter dimer cutting enzyme NheI‐HF (New England Biolabs) at 37°C for 2 h in a reaction volume of 15 μL. The adapter ligation step was performed immediately after the digestion with the adapters containing inline barcodes on both P5 and P7 adapters (Bayona‐Vásquez et al. 2019). Barcoded samples were equimolarly pooled and cleaned with 0.8X CleanPCR magnetic beads (GC biotech). A total of four libraries were prepared containing 132, 119, 55, and 32 samples each. Concentration was measured with Qubit 2.0 using the dsDNA HS assay (Life Technologies). The size selection step was performed with the Blue Pippin (Sage Science) using a 1.5% cassette and the R2 marker, targeting fragments between 230 and 320 bp. The libraries were submitted to a single‐cycle PCR to add the Tru5‐8 N primer, a unique molecular tag for each DNA fragment, followed by a 10‐cycle indexing PCR using a P5 short primer and P7 indexing primer to complete the library construction. The final library concentrations were determined using Qubit 2.0 dsDNA HS assay (Life Technologies) and checked in the Agilent 2100 sHigh Sensitivity DNA (Agilent Technologies) to analyze the length distribution and determine the size range of the DNA fragments in the library. The fragments were sequenced at the CCGA Kiel, Germany, using 150 bp paired‐end reads in one partial lane of the HiSeq 4000 platform (Illumina) with the TruSeq 300‐cycle Kit, aiming at 5 million reads per individual to yield a minimum of 20× coverage per individual sequenced.

### 2.4 ddRAD Analysis and Variant Detection

Illumina reads were demultiplexed based on P7 indexes, and the raw sequence quality was checked with Fastqc and Multiqc (Wingett and Andrews 2018) and preprocessed for quality as described in Driller et al. (2020). Adapter sequences were trimmed using Cutadapt (Martin 2011). P5 inline barcodes were demultiplexed using Flexbar (Dodt et al. 2012). PCR replicates were removed with the Python script Filter_PCR_duplicates.py, which recognizes identical combinations of iTru5_8N sequence tags and the first 100 bp of each read pair sequence and keeps only a single representative copy of the pair in the output. The software PEAR (Zhang et al. 2014) was used to merge read pairs with overlapping regions of at least 30 bp. All merged reads less than 250 bp were considered short fragments (i.e., out of the size selection range) and removed from the subsequent analysis. The unassembled reads were kept in the analysis as paired‐end reads, filtered for quality (*Q* > 30), and trimmed to a maximum length of 130 bp using Trimmomatic (Bolger et al. 2014). The checkestrictionsSitesPairedEnd.py script was used to check the presence of the CTAGA XbaI restriction site at the beginning of reads R1 and to check the presence of the AATTC EcoRI restriction site at the end of reads R2 and discard all the paired reads with incorrect restriction sites. Undigested and chimeric sequences were checked, and all reads containing intact XbaI and EcoRI restriction sites along the locus sequence were removed using the Filter_Reads.py script.

3RAD sequences were analyzed using the green turtle genome as a reference (RefSeq GCF_015237465.2, Bentley et al. 2023). Reads were mapped using the Bowtie2 software (Langmead and Salzberg 2012) with default parameters and the flags “‐no‐mixed” and “‐no‐discordant” to ensure that only paired reads aligned to the same locus would be present in the SAM files. Mapped reads were then analyzed with the Stacks reference‐based pipeline (Catchen et al. 2013). The gstacks.pl module was used to call variant sites within the population for each locus and to genotype each individual at each identified SNP. To filter population parameters, we used the populations.pl module. A locus was included in the final dataset only if it was genotyped in at least 60% of individuals within a population (‐r 0.6) and present in at least one population (‐p 1). The dataset was further processed to retain only SNPs by applying filtering criteria with VCFtools (Danecek et al. 2011). Variants were filtered based on: Depth of coverage (DP): A minimum depth of 10× and a maximum depth of 120X were required for both mean depth across samples and individual sample depths. Minor allele frequency (MAF): A threshold of 0.05 was applied to exclude low‐frequency variants. Missing data (MISS): SNPs with up to 20% missing data across samples (‐‐max‐missing 0.8) were allowed. The following command was used for VCFtools filtering: vcftools ‐‐vcf $VCF_IN ‐‐maf 0.05 ‐‐max‐missing 0.8 ‐‐min‐meanDP 10 ‐‐minDP 10 ‐‐max‐meanDP 120 ‐‐maxDP 120 ‐‐recode ‐‐stdout > filtered_output.vcf. This filtering strategy ensured the inclusion of high‐quality loci suitable for downstream analyses.

### 2.5 Kinship Analysis

The genetic similarity between individuals was investigated through a comprehensive analysis of all available SNPs. We employed several kinship analysis approaches, including five methods implemented in the COANCESTRY program within the related package in R (Wang 2011). These methods estimate pairwise relatedness between individuals using codominant molecular markers (Li et al. 1993; Lynch and Ritland 1999; Queller and Goodnight 1989; Ritland 1996; Wang 2002). Another analysis employed KING (Manichaikul et al. 2010). We also utilized Identity By State (IBS), a reliable predictor of identity by descent (IBD) with the appropriate SNP set (Powell et al. 2010). IBS was calculated using a custom Python script (main.py), which determined similarity values for each sample pair by averaging the proportion of shared alleles between genotypes across the entire SNP dataset. The similarity values were assigned as follows: 1 when two samples shared the same genotype, 0.5 when they shared one allele, and 0 when they had completely different alleles (i.e., opposite homozygotes). To enhance interpretability, we normalized the IBS values (normalized‐IBS), scaling them to range from 0 (unrelated) to 1 (identical). The normalization process defined 1 as the maximum identity (same sample) and 0 as the average IBS value between the least related families or nests in the dataset. The normalized‐IBS value for each sample pair was calculated using the formula: Normalized‐IBS = (IBS value − av. min)/(1 − av. min), where av. min is the average IBS value between all individuals comprising the two most distinct families or nests.

Thus, the normalized IBS removes the overall levels of identities that are found by chance between any two individuals coming from a common gene pool.

The statistical analyses examined pairwise values across various groups, including families, nests, locality, and haplotypic groups. Pairwise values are not statistically independent; thus, we used computer simulations for calculating the confidence intervals wherever necessary. For each comparison, 1000 simulations were performed, each consisting of a random permutation of all participating individuals or groups. Every permutation was randomly divided into groups containing the same number of group members, and the average within‐group value was calculated. From these 1000 simulated averages, we obtained an estimated 95% confidence interval and compared it to the observed within‐group values, verifying the significance of each result.

Other genetic data analyses were conducted using the SambaR package in R (Jong et al. 2021). Each VCF file was converted into the PED/MAP format using PLINK (Purcell et al. 2007), a widely used tool in genetic analysis. The resulting files were then corrected to associate sample names with population names based on a predefined order using a population list. Finally, the PED/MAP files were converted into the RAW/BIM format using PLINK. The data was then uploaded and processed using the SambaR package. Population structure analysis was conducted using the *findstructure* command in SambaR to facilitate a comprehensive exploration of genetic relationships and to detect genetic partitions in samples with the same STR haplotype. The implemented multifaceted approach included the construction of Principal Coordinates Analysis (PCoA), for genotype proportion visualization and assessment of heterozygosity proportion.

## 3 Results

### 3.1 mtDNA Analysis

**FIGURE 1:**
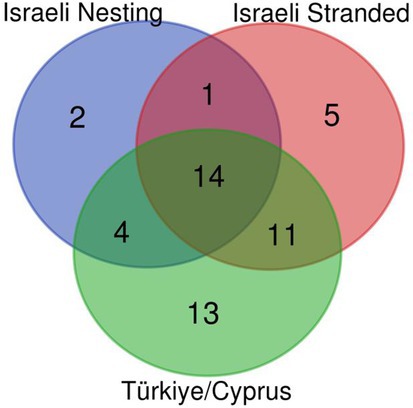
Venn diagram describing the distribution of Mediterranean mtDNA haplotypes among three categories: Israeli nests (blue), Israeli stranded (red) and Türkiye or Cyprus nests (green).

A total of 50 Mediterranean mtDNA haplotypes were recorded from this and previous studies (Karaman et al. 2022; Tikochinski et al. 2018), and are presented in Table S2. Of these, 42 haplotypes were found in the Turkish and Cypriot nesting populations. Three haplotypes were unique to the Israeli nests, while five more haplotypes were found in stranded turtles along the Israeli shore. The Israeli nesting population consists of 21 haplotypes, while the total of nesting and stranded Israeli turtles reaches 37. Figure 1 summarizes the current records obtained for the different haplotypes in the Mediterranean divided into three categories: Türkiye/Cyprus nests, Israeli nests, and Israeli stranded turtles. When stranded haplotypes are included, the Israeli population represents 74% of the total Mediterranean haplotypes.

Table S1 provides detailed information on all 269 samples analyzed in this study, including mtDNA haplotypes, nesting dates, and locations. It also specifies the sample sources (e.g., stranded adults, dead hatchlings, or breeding stock members). Among the 10 stranded turtles and one nesting female, 12 different mtDNA haplotypes were identified, as indicated in Table S1. To identify females sharing the same haplotype, we focused on hatchling samples representing four haplotypes observed across multiple nests along the Israeli coast. Among these, three haplotypes (6‐8‐8‐4, 6‐9‐6‐4 and 8‐7‐7‐4) are the most prevalent in Israel, while 6‐8‐9‐4 was identified as a heteroplasmic minor allele of 6‐8‐8‐4 (Tikochinski et al. 2018, 2020). Additionally, one hatchling with the 8‐7‐7‐4 haplotype, unique to Israel, was included in the analysis. Hatching locations and dates, when known, are provided in Table S1 and Figure S1.

### 3.2 RADseq Analysis

The 3RAD data yielded an average of 4,634,968 raw reads per individual, with 42% retained after preprocessing (Table S3). Filtered reads were utilized for loci construction and SNP calling using the Stacks pipeline. The resulting SNPs had an average coverage of 40× (STDEV = 17). Out of the 269 samples, 10 did not pass the coverage filter and were excluded from further analysis. In total, five distinct datasets were processed, generating five separate VCF files. Table S4 provides details on the number of loci and SNPs retained at each filtering step for each dataset.

### 3.3 Breeding Stock

**FIGURE 2:**
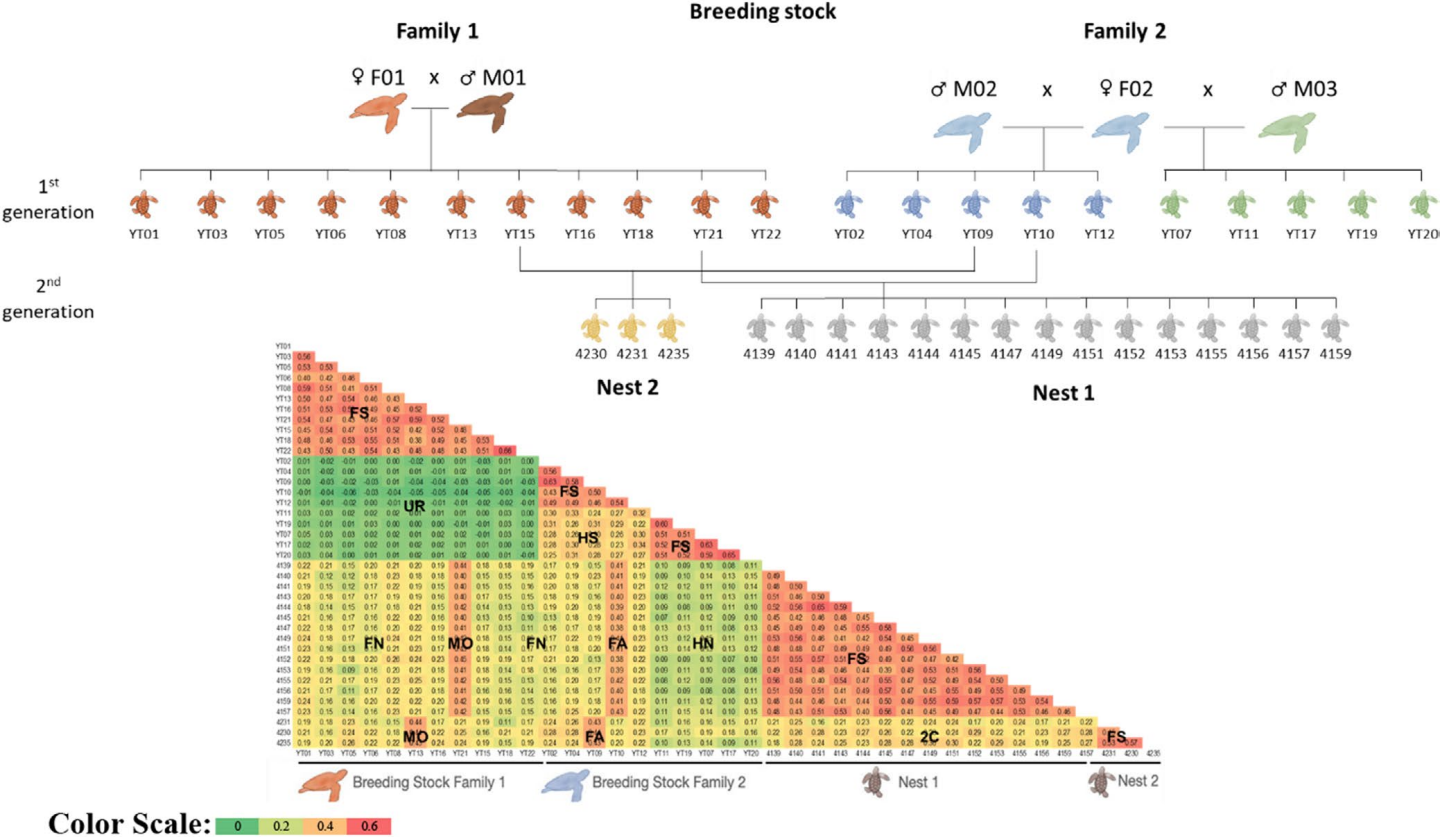
Normalized‐Identity by State (IBS) values within the breeding stock and the first two nests of the breeding stock. 2C, two‐way cousins; FA, father; FN, full nephews; FS, full‐siblings; HN, half‐nephews; HS, half‐siblings; MO, mother; UR, unrelated. Color scale from high values of genetic identity (red) to low values (green).

We conducted a Principal Coordinate Analysis (PCoA) for the two families (1 and 2) within the breeding stock (Figure S2). The results showed that Family 1 formed a single, cohesive cluster, while Family 2 displayed two distinct clusters, suggesting the presence of two different fathers. We then used 12,795 informative SNPs, proportionally distributed across the entire genome according to chromosome length, to establish an Identity by State (IBS) heatmap among all the samples, capturing various degrees of kinship (Figure 2 and Table 1). Family 1 consisted of 11 hatchlings with normalized‐IBS values averaging 0.45 ± 0.04 among all of them, indicating that they are full‐siblings. Family 2 consisted of 10 individuals clearly divided into two groups of five full‐siblings with average values of 0.47 ± 0.05 and 0.50 ± 0.04. The average normalized‐IBS values between the two groups were 0.28 ± 0.02. Since they came from the same nest, we concluded that they are half‐siblings.

**TABLE 1:** Normalized‐Identity by State (IBS) values between full‐siblings, half‐siblings, hatchlings and parents, full nephews, half‐nephews, two‐way cousins, and unrelated individuals as calculated by the breeding stock of two families and their two nests. Average and standard deviation (SD) are indicated.

	Full‐siblings	Half‐siblings	Hatchlings and mother	Hatchlings and father	Full‐nephews	Half‐nephews	Two‐way cousins	Unrelated
Family 1	0.49							0.00
Family 2								0.00
Family 2a	0.51	0.28						
Family 2b	0.55	0.28						
Nest 1	0.50		0.42	0.40	0.18	0.11	0.24	
Nest 2	0.52		0.43	0.43	0.21	0.14	0.24	
Average	0.50	0.28	0.42	0.41	0.19	0.11	0.24	0.00
SD	0.05	0.03	0.01	0.02	0.03	0.03	0.04	0.02

To better determine kinship differences, we included in the analysis the hatchlings from Nest 1 and Nest 2, representing the second generation of the breeding stock's genetic composition, and calculated the normalized‐IBS (Figure 2). Nest 1 and Nest 2 each consist of full‐siblings, exhibiting normalized‐IBS values of 0.45 ± 0.04 and 0.47 ± 0.04, respectively, which are comparable to those observed in the previous generation. Table 1 presents the average values representing the same degree of kinship, including full‐siblings, half‐siblings, hatchlings versus mother, hatchlings versus father, full‐nephews, half‐nephews, two‐way cousins, and unrelated individuals. The advantage of using the IBS approach over the other common kinship analysis methods is demonstrated in Table S5 and explained in the discussion.

### 3.4 Nesting Population

**FIGURE 3:**
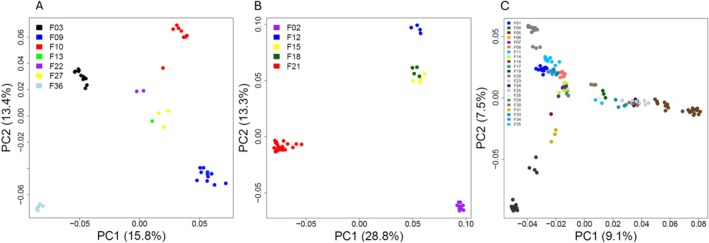
Principal coordinate analysis (PCoA) graphically representing a resemblance matrix between hatchlings of the Israeli nesting females (F01–F36). (A) Hatchlings with mtDNA haplotype 8774, (B) Hatchlings with mtDNA haplotype 6964, (C) Hatchlings with mtDNA 6884 + 6894 haplotypes.

**FIGURE 4:**
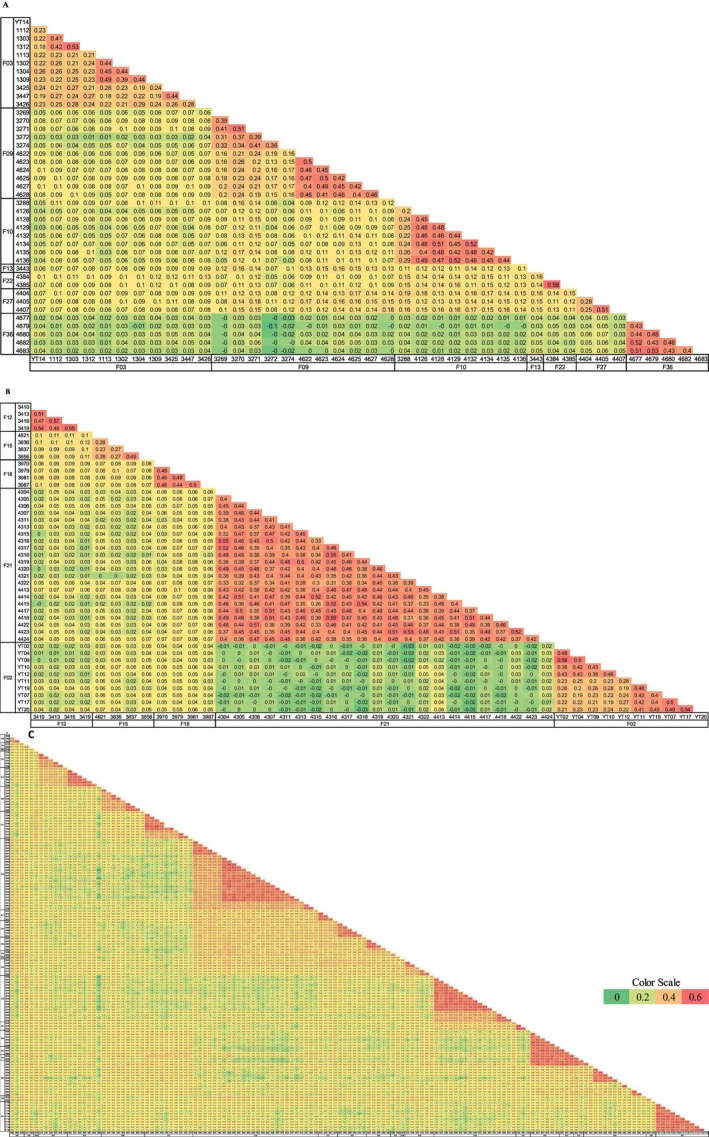
Normalized‐Identity by State (IBS) values for females' hatchlings sharing (A) The 8774 mtDNA haplotype. (B) The 6964 mtDNA haplotype. (C) The 6884 and 6894 mtDNA haplotypes. Color scale from high values of genetic identity (red) to low values (green).

We used the 259 informative samples to construct a normalized‐IBS analysis matrix, providing a comprehensive overview of the nesting population and enabling comparisons with the nine stranded samples (Figure S3). To better distinguish between nesting females sharing the same mtDNA haplotype and accurately estimate the number of nesting females, we conducted three separate analyses. These analyses focused on hatchlings from: (1) eight females' hatchlings with the 8‐7‐7‐4 haplotype, (2) five females' hatchlings with the 6‐9‐6‐4 haplotype, and (3) 22 females' hatchlings with the 6‐8‐8‐4 or the 6‐8‐9‐4 haplotype. Principal Coordinate Analysis (PCoA) (Figure 3) and the normalized‐IBS matrix (Figure 4) were constructed for these groups. One sample of a hatchling with the 8‐7‐7‐4 haplotype was added to serve as an unrelated sample control.

By combining nests' locations and dates with the normalized‐IBS matrix, we identified half‐siblings within nests resulting from polyandry, thereby determining the actual number of males (fathers). Notably, in one instance, half‐siblings with different mtDNA haplotypes were linked to a shared father. In total, we detected 60 mating events involving 35 potential females: 19 with the 6‐8‐8‐4 haplotype, 3 with 6‐8‐9‐4, 5 with 6‐9‐6‐4, and 7 with the 8‐7‐7‐4 mtDNA haplotype (Figure 4). One female with the 8‐7‐8‐4 haplotype was involved in one mating event. Considering that this study focused on only 5 of the 21 Israeli haplotypes, the minimum estimate of nesting females rises to 51, substantially higher than previous estimates based solely on mtDNA haplotypes. Additionally, for the first time, we investigated male contribution, identifying 59 potential fathers among the Israeli hatchlings analyzed in this study alone.

By tracking the different potential mothers, we could observe their breeding habits. Table 2 lists mothers with recurrent nesting years, showing a frequency of 3–4 years between laying seasons. Notably, one potential father (M28) participated in two mating events, 3 years apart, with nests located in the same areas. The intervals between nests laid by the same female in the same year, indicated in Table S1, averaged 14 days, with a minimum of 8 days and a maximum of 23 days.

**TABLE 2:** Intervals between years for recurrent nesting females or mating males.

Potential parent	Intervals between years
F03 (Sample 182)	2004, 2008, 2013^a^, 2017
F04	2013, 2020
F05	2013, 2016
F07	2013, 2020
F08	2014, 2017, 2021
F09	2016, 2021
F10	2016, 2019
F11	2017, 2020
F15	2018, 2021
F16	2018, 2021
M28	2018, 2021

^a^ Observed and sampled when nested (no dead hatchlings).

Since not all the nests are sampled (as in nests containing no dead hatchlings) we cannot know whether a particular female had another nesting event, which could shorten the interval. We have also recorded and calculated the geographical distances between nests that were laid by the same female in the same season and along the years (Table S1). This enabled the assessment of the females' philopatric behavior.

**FIGURE 5:**
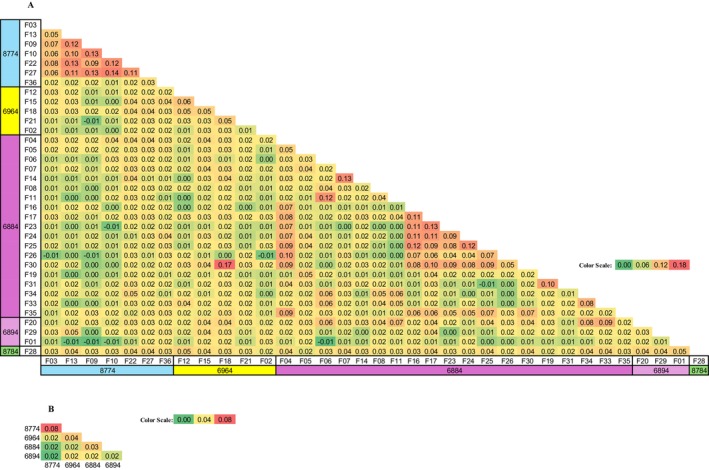
Mean normalized‐Identity by State (IBS) (A) between the hatchlings of each of the 35 females. (B) Between hatchlings sharing the same mtDNA haplotype. Color scale from higher values of genetic identity (red) to lower values (green).

We then looked at the normalized‐IBS for the hatchlings of the different potential mothers (Figure 5A). We found no significant differences between the hatchlings of mothers sharing the same or different haplotypes. The only exception was the 8‐7‐7‐4 hatchlings, with an average normalized‐IBS of 0.08, significantly higher than the normalized‐IBS of 0.02 between them and hatchlings with the other three haplotypes (Figure 5B).

### 3.5 Israeli Stranded Turtles

**FIGURE 6:**
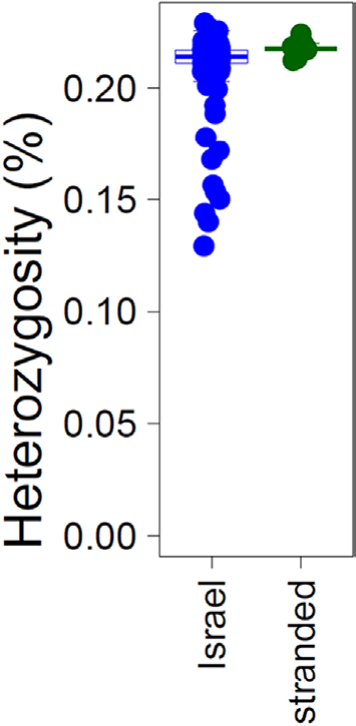
Heterozygosity levels (percentage of heterozygote sites) of all samples. Blue—Israeli nesting population, Green—stranded turtles.

Out of 259 samples, nine turtles were stranded and brought to a rescue center due to stress or discomfort. There was no significant difference in average normalized‐IBS between the stranded turtles or between them and the local hatchlings (Figure S3). Additionally, the average heterozygosity between the stranded turtles and local hatchlings showed no significant difference (Figure 6), aligning with previous studies comparing Mediterranean and Atlantic green turtles (Bentley et al. 2023). A small proportion of the local hatchling population, which showed lower heterozygosity levels, had a higher proportion of missing data.

## 4 Discussion

The Mediterranean sea turtle population, particularly in Israel, experienced a significant decline in numbers and genetic variability until the 1930s. To support the declining green turtle population, INPA established the Israeli breeding stock, initially without genetic assistance and with variation considered only by mixing hatchlings from two geographically remote locations (Table S1). Early 21st‐century research based on mtDNA SNP haplotyping showed little variation within the Mediterranean population (Bowen et al. 1992 and many more). However, the introduction of mtDNA STR haplotyping revealed greater genetic diversity within the Israeli population (Tikochinski et al. 2012), later highlighting its importance as one of four separate management units in the region (Tikochinski et al. 2018). A more recent study (Karaman et al. 2022) examining 12 nesting populations concluded that the Israeli population shows no genetic differentiation from the nesting sites in eastern Turkey and North Cyprus, while also exhibiting the highest genetic diversity among them. Satellite telemetry‐supported studies on habitat utilization and migratory corridors by green turtles in the Mediterranean, many of which nest in Turkey and north Cyprus, pass along the Israeli coast (Godley et al. 2002; Rees et al. 2008; Stokes et al. 2015; Levy et al. 2017), further emphasize the importance of the Israeli population.

Relying solely on maternally inherited mtDNA for population analysis is extremely limited. Firstly, it does not allow us to distinguish between females that share the same mtDNA haplotype. Secondly, it provides no information about the male contribution to the population. One of the most important goals of the breeding program was to expand the breeding stock by recruiting more individuals with a desirably distant genetic background. Selection by mtDNA haplotyping only is insufficient since relatives like half‐siblings can also have different haplotypes. A more comprehensive view of genetic variation is needed to establish a breeding stock with maximum variability (Alvarez‐Estape et al. 2022; Ortego et al. 2024). Several studies have employed microsatellite markers to investigate the mating habits of various sea turtle species, with a particular focus on multiple paternity and male contributions (Barbanti et al. 2022; Jensen et al. 2006; Lasala et al. 2013; Shamblin et al. 2017; Theissinger et al. 2008). These studies utilized 4–18 polymorphic loci of unknown chromosomal localization and relied on DNA from both mothers and their hatchlings to reconstruct male contributions. Advanced genomic approaches, such as RADseq, have since enabled the use of thousands of markers distributed across the genome. Furthermore, the recently published genome of a local green turtle from the breeding stock (Bentley et al. 2023) has provided a more comprehensive understanding of the genome‐wide genetic diversity. The extensive number of evenly distributed SNP markers across the genome facilitates direct genetic comparisons between any two samples within a dataset, enabling robust and accurate kinship determination.

### 4.1 Kinship Analysis

We explored various approaches to determine kinship. Clustering methods typically utilize only a subset of the total genetic variability to create two‐dimensional visualizations, as reflected in Figure 3 (PCoA), where hatchlings from different females often cannot be distinguished, particularly when multiple nests are analyzed together.

To address this limitation, we examined several kinship analysis methods that leverage the complete SNP dataset within a given analysis, focusing on the breeding stock dataset of 12,795 SNPs (Table S5). All these approaches successfully differentiated full‐siblings from half‐siblings. Among them, normalized‐IBS proved particularly effective for distinguishing more distant relatives, such as nephews and cousins. For all kinship degrees, the normalized‐IBS had the lowest Coefficient of Variation (CV). This robustness also enhances its ability to differentiate full‐siblings from half‐siblings in scenarios where the parents involved are more genetically similar. Therefore, we decided to proceed with the normalized‐IBS method for all kinship analyses in this study.

Our normalized‐IBS values are calculated relative to the non‐related sample values within each dataset. As a result, the same pair of samples can yield slightly different normalized‐IBS values depending on the dataset context. However, since the values are merely scaled for normalization, the relative differences between samples remain consistent.

The normalized‐IBS values for full‐ and half‐siblings can vary depending on the genetic differences between their parents (i.e., how unrelated the parents are) and the likelihood of gene sharing between siblings. As such, there is no universal threshold to distinguish full‐siblings from half‐siblings. These differences are evident in the spread of values observed for sibling relationships. The accuracy of relatedness estimation between females will therefore depend on the number of hatchlings included in the analysis.

The robustness of the normalized‐IBS approach is evident in Figure S3. Across all nests, the average normalized‐IBS value for full‐siblings is 0.441, with a standard deviation (SD) of 0.028 and a coefficient of variation (CV) of 0.12. For half‐siblings, the average value is 0.213, with an SD of 0.027 and a CV of 0.13. These values remain highly consistent across the five datasets analyzed in this study. However, using datasets with a smaller number of individuals improves accuracy, as demonstrated for the breeding stock and their offspring in Table S5. The breeding stock dataset, which includes 12,795 SNPs (S5A), proves to be more accurate than the full dataset of 18,119 SNPs (S5H). While the differentiation between full and half‐siblings remains robust in both datasets, the breeding stock dataset provides slightly better resolution for detecting lower degrees of kinship.

### 4.2 Breeding Stock

As the breeding stock is composed of two families already reproducing, it presented a unique opportunity to study kinship and genetic similarity. The very evident difference between the full and half‐siblings normalized‐IBS values, a true indication of Identity by Descent (IBD), was used for every further dataset analysis where known siblings (obtained from the same nest), as well as unrelated individuals, were present. Even though we only needed to differentiate between full‐ and half‐siblings in the nesting population analysis, normalized‐IBS values for other degrees of kinship have also been calculated (Table 1) demonstrating the accuracy of the method, especially for assessment of unrelated individuals.

A noteworthy finding revealed that the average normalized IBS values between the full‐siblings of nests 1 and 2 were significantly higher than those between the siblings and their parents. This pattern reflects the genetic distance between the parents: the more genetically distant two parents are from each other, the closer their offspring will be to each other compared to their parents. This observation highlights the substantial genetic variability present among the breeding stock founders.

The low normalized‐IBS values observed between the breeding stock's two families, compared to all unrelated samples regardless of haplotype, underscore their distinct genetic backgrounds. Additionally, the genetic distances between the half‐siblings in family 2 indicate that the two fathers are unrelated. Despite the limited number of founders, the breeding program began with five genetically distinct individuals. Nevertheless, the breeding stock should be fortified with as much genetic variability as possible.

### 4.3 Nesting Population

A major goal of this study was to better evaluate the size of the Israeli green turtle population, especially nesting females.

We have analyzed 259 Israeli hatchlings, sharing four haplotypes commonly present in the local nests' mtDNA. Three of these haplotypes are abundant throughout the Mediterranean (6‐8‐8‐4, 6‐8‐9‐4, and 6‐9‐6‐4), while one (8‐7‐7‐4) is mainly found in Israeli nests (Tikochinski et al. 2018). One sample of a hatchling with the 6‐7‐8‐4 was added to this survey and served as a control for a potential unrelated sample. The normalized‐IBS heatmap of all 259 samples shows the method's power, with full‐siblings easily detected (average IBS value of 0.471 ± 0.028 across all nests, Figure S3). The collection of hatchlings from various nests also enabled the detection of half‐siblings within the same nest or another nest (average normalized‐IBS value of 0.240 ± 0.026).

In total, we detected 60 mating events involving 35 potential females and 59 different potential males (Table S1). The contribution of males to mating events in green turtles has been investigated using genomic STR markers. In the most extensive study to date, examining a 50‐year reintroduction program of green turtles in the Cayman Islands, a balanced female‐to‐male ratio of 1:1 was observed (Barbanti et al. 2022). Similar studies in the Mediterranean have reported ratios of 1:1.3 or 1:1.4, favoring males (Wright, Fuller, et al. 2012; Wright, Stokes, et al. 2012). In our study, we identified a higher ratio of 1:1.68. However, this value may be an overestimation, as our analysis was limited to a selected group of females (only 4 haplotypes) without imposing restrictions on the males. This suggests that the observed ratio might decrease with a broader sampling approach. Unlike the studies mentioned above, which included DNA from both hatchlings and their mothers, our approach represents the first analysis based solely on hatchlings' DNA to identify both their mothers and fathers.

This study identified 35 females sharing five haplotypes. When incorporating the 16 additional haplotypes previously identified in Israeli nests over the past 20 years but not included in this study, the total minimum number of nesting females increases to 51. This figure is significantly higher than previous estimates provided by the Mediterranean sea turtle conservation community (Karaman et al. 2022; Levy and Rybak 2022; Tikochinski et al. 2018). This number is likely an underestimate, as not all nests are sampled. Our dataset consists exclusively of DNA extracted from dead hatchlings collected by INPA rangers, representing approximately 5% of the nests laid during the study period (Levy and Rybak 2022). Improved monitoring efforts and more extensive sampling could reveal additional nesting females, independent of their mtDNA haplotypes.

The mtDNA haplotyping proved to be a poor indicator of kinship. We found similar average normalized‐IBS values between hatchlings of mothers sharing the same or different haplotypes (Figure 5). Maternal lineage can maintain the same haplotype for many generations among distant relatives. An exception was haplotype 8‐7‐7‐4, which showed significantly higher normalized‐IBS values, indicating the genetic proximity of an Israeli unique haplotype shared by closely related females. Another exception was the similarity between hatchlings of two females (F18 and F30) with different mtDNA haplotypes due to a common father (M28). This further emphasizes the importance of IBS analysis.

Our RADseq data enabled genetic verification of documented breeding habits of female green turtles. Previous studies showed that female green sea turtles generally nest every 2–4 years (Casale 2010), and we found intervals of 3–4 years (Table 2). The only male (M28) whose philopatry we identified was detected after an interval of 3 years (Table 2), which falls at the upper limit of previously estimated Mediterranean male breeding intervals (Wright, Fuller, et al. 2012; Wright, Stokes, et al. 2012).

Previous studies also indicated that during a nesting season, a single female may mate with several males, store sperm, and lay multiple clutches of eggs (Bowen et al. 1992). Multiple paternity was found in 10 out of the 84 sampled nests, many of which had one or very few hatchlings. When considering only the nests with more than 5 hatchlings, 6 out of 18 (one third) present multiple paternity. Additionally, it was observed that, on average, a female lays between three and five clutches per season, with intervals of about 2 weeks between each nesting event (Casale 2010). Our study revealed, despite sampling limitations, a few females with 3–4 clutches per year, with intervals ranging from 8 to 22 days (Table S1). The higher value is not conclusive and may split into two intervals since not all nesting events are documented.

Green turtle females are known for their natal homing (Lohmann and Lohmann 2019). Our findings shed light on the degree of natal homing and reveal that although most nests laid by the same female are found in proximity, they can occasionally nest up to 63.1 km away in the same season and even 90 km away in multiple seasons (Table S1).

The analysis of runs of homozygosity (RoHs) in the reference genome has provided evidence of past inbreeding events within the Israeli green turtle population (Bentley et al. 2023). However, our study's findings suggest that direct inbreeding is not currently detectable within the 60 documented mating events. The average normalized‐IBS values among full‐siblings across all nests exhibit low variation (0.49 ± 0.04), with no exceptionally high values indicative of recent inbreeding. Similarly, the normalized‐IBS values of half‐siblings (0.21 ± 0.03) further support that the parents involved are not direct relatives.

These results imply that while historical inbreeding events may have occurred, the current population shows no direct signs of mating between close relatives within our sampled dataset. Nevertheless, the absence of detectable inbreeding does not rule out the possibility of second‐ and third‐degree kinship among mating individuals. This underscores the importance of refining methods to detect more distant kinship relationships, which could shed light on the genetic structure and dynamics of the population.

Future studies should employ genome‐wide analyses and include a larger number of hatchling samples from different nesting females to improve the resolution of these analyses. Such efforts will provide a more comprehensive understanding of the genetic diversity, breeding patterns, and long‐term viability of this endangered population.

### 4.4 Stranded Turtles

Nine of the stranded turtles in this survey were candidates for the breeding stock. Previously, mtDNA haplotypes were used to evaluate these candidates, but determining a young green turtle's sex before the age of 10 is very difficult. Additionally, half‐siblings with a shared father are not desirable for the breeding stock. Our approach provides an accurate evaluation of kinship, aiding rational decision‐making for populating the rescue center's breeding stock.

All the candidates showed very low normalized‐IBS values when compared to the Israeli nesting population samples, including the two with similar mtDNA haplotypes (6‐8‐8‐4 and 6‐9‐6‐4). These candidates were as genetically distant from the samples with the same haplotype as they were from those with different haplotypes. Therefore, we can consider their suitability for the breeding stock based on other important factors like maturity and sex.

## 5 Conclusion

Following many years of conservation efforts, the Mediterranean sea turtle populations are showing signs of improvement, especially among loggerhead turtles (Stokes et al. 2015). Our genomic‐assisted evaluation of the Israeli green turtle population indicates similar positive trends. We plan to expand our survey to assess the status of the entire Mediterranean population.

The Israeli rescue center's breeding stock is currently the only active one in the Mediterranean. Significant time and effort have been invested in its inception and operation. Using our genomic approach, we aim to maximize the genetic contribution to the Mediterranean population and monitor its changes and welfare in the coming years.

## Author Contributions

**Talya Ohana:** data curation (equal), formal analysis (equal), software (supporting), writing – original draft (supporting), writing – review and editing (supporting). **Larissa S. Arantes:** formal analysis (supporting), software (supporting). **Gili Tikochinski:** data curation (supporting), methodology (supporting), software (equal). **Reut Cohen:** data curation (supporting). **Olga Rybak:** data curation (supporting). **Adi Gaspar:** software (supporting). **Sarah Sparmann:** data curation (supporting). **Susan Mbedi:** data curation (supporting). **Yaniv Levy:** data curation (supporting), writing – review and editing (supporting). **Camila J. Mazzoni:** data curation (supporting), methodology (supporting), software (supporting), writing – original draft (supporting), writing – review and editing (supporting). **Yaron Tikochinski:** conceptualization (lead), data curation (lead), formal analysis (lead), funding acquisition (lead), methodology (lead), project administration (lead), writing – original draft (lead), writing – review and editing (equal).

## Conflicts of Interest

The authors declare no conflicts of interest.

## Supporting information


Data S1

## Data Availability

All data has been made available on the NCBI Sequence Read Archive (SRA) database under BioProject PRJNA1145648. All scripts used in the ddRAD analysis are available at https://github.com/Gilitiko/RAD‐Seq‐Scripts. Other relevant data (e.g., VCF files, Supporting Information) are available on Figshare: https://figshare.com/s/f06b760480518f5f3521.
